# Neurogenetic mechanisms of risk for ADHD: Examining associations of polygenic scores and brain volumes in a population cohort

**DOI:** 10.1186/s11689-023-09498-6

**Published:** 2023-08-31

**Authors:** Quanfa He, Taylor J. Keding, Qi Zhang, Jiacheng Miao, Justin D. Russell, Ryan J. Herringa, Qiongshi Lu, Brittany G. Travers, James J. Li

**Affiliations:** 1 Department of Psychology, University of, Wisconsin-Madison, 1202 W. Johnson Street, Madison, WI 53706 USA; 2 Waisman Center, University of Wisconsin-Madison, Madison, USA; 3 Department of Psychology, Yale University, New Haven, USA; 4 Department of Educational Psychology, University of Wisconsin-Madison, Madison, USA; 5 Department of Biostatistics and Medical Informatics, University of Wisconsin-Madison, Madison, USA; 6 Department of Psychiatry, School of Medicine and Public Health, University of Wisconsin, Madison, USA; 7 Center for Demography of Health and Aging, University of Wisconsin-Madison, Madison, USA; 8 Department of Statistics, University of Wisconsin-Madison, Madison, USA; 9 Department of Kinesiology, University of Wisconsin-Madison, Madison, USA

**Keywords:** ADHD, Polygenic scores, Brain volume, Functional annotation, Multiple mediation

## Abstract

**Background:**

ADHD polygenic scores (PGSs) have been previously shown to predict ADHD outcomes in several studies. However, ADHD PGSs are typically *correlated* with ADHD but not necessarily reflective of *causal* mechanisms. More research is needed to elucidate the neurobiological mechanisms underlying ADHD. We leveraged functional annotation information into an ADHD PGS to (1) improve the prediction performance over a non-annotated ADHD PGS and (2) test whether volumetric variation in brain regions putatively associated with ADHD mediate the association between PGSs and ADHD outcomes.

**Methods:**

Data were from the Philadelphia Neurodevelopmental Cohort (*N* = 555). Multiple mediation models were tested to examine the indirect effects of two ADHD PGSs—one using a traditional computation involving clumping and thresholding and another using a functionally annotated approach (i.e., *AnnoPred*)—on ADHD inattention (IA) and hyperactivity-impulsivity (HI) symptoms, via gray matter volumes in the cingulate gyrus, angular gyrus, caudate, dorsolateral prefrontal cortex (DLPFC), and inferior temporal lobe.

**Results:**

A direct effect was detected between the *AnnoPred* ADHD PGS and IA symptoms in adolescents. No indirect effects via brain volumes were detected for either IA or HI symptoms. However, both ADHD PGSs were negatively associated with the DLPFC.

**Conclusions:**

The *AnnoPred* ADHD PGS was a more developmentally specific predictor of adolescent IA symptoms compared to the traditional ADHD PGS. However, brain volumes did not mediate the effects of either a traditional or *AnnoPred* ADHD PGS on ADHD symptoms, suggesting that we may still be underpowered in clarifying brain-based biomarkers for ADHD using genetic measures.

## Background

Attention-deficit/hyperactivity disorder (ADHD) is a neurodevelopmental disorder that affects 3–6% of youths and adults worldwide [1, 2]. Genes explain a substantial proportion of the variance in ADHD [3], accounting for an estimated 70 to 80% of its variation [4, 5]. Genome-wide association (GWA) studies have identified several key genetic variants associated with ADHD [6, 7], but it is widely believed that there are many genes of individually small effects that contribute to its etiology [3, 8, 9]. Polygenic scores (PGSs) are typically used to quantify a person’s polygenic risk for a trait of interest [10]. PGSs are typically computed as the sum of the number of risk alleles at each genetic locus weighted by their effect sizes, which are informed by summary statistics of a GWA study. According to multiple meta-analyses, ADHD PGSs explain approximately 4% of the variance in ADHD outcomes across studies and populations [3, 9]. By comparison, the most statistically significant genetic variant identified in the ADHD GWA study accounted for a miniscule .1% of the variance in ADHD [6].

ADHD PGSs correlate with ADHD outcomes across many studies because the genetic effect sizes that are used in their computations are based entirely on the statistical associations (usually as regression betas or odds ratios) between individual level genotypes and a trait of interest conducted in a large GWA study. However, PGS associations with trait outcomes may not necessarily reflect casual (e.g., neurobiological) signals [3]. This knowledge is needed to help elucidate unique neurobiological mechanisms for ADHD, which can then contribute to the development of novel therapeutic targets. Notably, ADHD GWA studies already provide valuable biological information, but this information has rarely been leveraged in ADHD PGS studies. For instance, a functional annotation of the genetic variants associated with ADHD showed significant overrepresentation in regulatory regions of DNA in cells in the anterior caudate, cingulate gyrus, angular gyrus, dorsolateral prefrontal cortex (DLPFC), and inferior temporal lobe, suggesting that ADHD-associated variants may be involved in the regulation of gene expression in these five brain regions and influence their structure and function [6]. Furthermore, several of the GWA-associated ADHD genes (e.g., *SORCS3*,* DUSP6*,* SEMA6D*) are involved in neurotransmission, neuronal development, and neuronal plasticity. Despite the wealth of neurobiological information gained from powerful ADHD GWA studies, these data have not been incorporated in ADHD PGS applications.

One powerful approach to do so is called *AnnoPred*, a statistical method that upweights genetic variants located in annotation categories that are overrepresented in a GWA study of interest [11]. Annotation categories include enhancers, promoters, conserved regions, coding regions, transcription factor binding sites, and cell-type specific epigenomic marks in the central nervous system, immune system, cardiovascular system, gastrointestinal system, and skeletal muscles. Upweighted genetic variants are considered more functionally relevant (by effect size) for a trait of interest. The *AnnoPred* PGS method demonstrated superior accuracy in complex trait predictions compared to other PGS methods [11]. Thus, methods such as *AnnoPred* should not only improve ADHD PGS prediction performance over a non-annotated ADHD PGS, but it should also provide more signal for elucidating potential neurobiological pathways of risk for ADHD given that *AnnoPred* PGS specifically upweights functionally relevant signals.

In addition to gene enrichment findings from ADHD GWA studies [6], other possible brain-based mechanisms underlying the risk for ADHD come from the broader neuroimaging literature [12, 13]. Several studies have reported associations between smaller regional volumes in areas such as the caudate, prefrontal cortex, temporal lobe, cingulate cortex, and cerebellum in samples of children and adolescents with ADHD compared to controls [14–17]. In particular, the caudate and dorsolateral prefrontal cortex have been extensively implicated in ADHD studies [18–21]. The caudate nucleus is involved in motor movement, associative learning, memory, and goal-directed behavior and was found to be coactive with the DLPFC and anterior cingulate cortex during a range of cognitive and motor tasks [22]. The DLPFC is linked to higher level cognitive function such as inhibition, planning, and working memory [23]. Significantly smaller caudate nucleus and DLPFC have been documented in youths with ADHD relative to typically developing youths [16, 19]. Other studies have shown that brain volume differences between children with ADHD and controls are more dispersed over the brain than previously hypothesized. For example, several studies have reported volumetric reductions in the temporal lobe and cingulate cortex in youths with ADHD relative to controls [15, 20, 24, 25].

Whereas prior studies investigated genetic and brain volume associations with ADHD as separate mechanisms, Alemany and colleagues [26] examined whether brain volume might *mediate* genetic associations (via PGSs) of several psychiatric traits and disorders, including schizophrenia, depression, bipolar disorder, ADHD, and autism. This study examined the association between PGSs for five psychiatric disorders and volume of several brain regions of interest (i.e., amygdala-hippocampus complex, caudate, putamen, thalamus, cerebellum, total brain, ventricles, cortical and subcortical gray matter, and total white matter) in a large, population-based sample of 9–11-year-old children (*n* = 1139). ADHD PGSs were associated with smaller caudate volume but not the other regions, and the association between the ADHD PGS and male-specific attention problems was mediated by caudate volume, in line with the well-replicated association between caudate and ADHD. Their findings provide compelling (albeit still limited) evidence that ADHD PGSs may more proximally tap into biological signals in the development of ADHD.

However, one critical limitation of the prior study [26] is that development was not accounted for. ADHD symptom presentations vary depending on the age of the individual [27, 28]. For instance, while inattention (IA) symptoms tend to be relatively stable throughout development, hyperactivity/impulsivity (HI) symptoms tend to decline by adulthood [27, 29]. Thus, volumetric brain differences between ADHD and control samples may be age-dependent. In a mega-analysis of 23 cohorts of ADHD cases and cohorts (*n* = 3200), researchers found that volumetric differences between groups were larger among children (aged between 4 and 14) than among adolescents (aged between 15 and 21) or among adults (aged 22–63) [30]. Specifically, they found that accumbens, amygdala, caudate, hippocampus, putamen, and total intracranial volume were smaller in youths with ADHD than in controls, whereas only the hippocampus was significantly smaller in adolescents with ADHD relative to controls; no brain volume differences were found in adults with ADHD compared to controls. Another study compared whole-brain volume and subcortical regional volume between individuals with ADHD and controls in a Dutch sample of youths and adults (*n* = 672). They reported a reversal in the direction of difference between cases and controls among young adolescents (aged 8–15), older adolescents (aged 16–22), and young adults (aged 22–30), such that caudate and putamen volume were smaller in adolescents with ADHD than controls, but larger in young adults with ADHD than controls [31]. The more restricted and younger age range of the adult sample in the latter study [31] likely contributed to the inconsistent findings compared to the first study [30]. In light of these inconsistent findings, developmental considerations should be taken into account in genetically-informed studies of ADHD.

This current study focused on children (aged 8–11), adolescents (aged 12–17), and young adults (aged 18–21) from the Philadelphia Neurodevelopmental Cohort (PNC). Although ADHD PGSs are frequently used in clinical samples, they are also predictive of psychiatric traits in population samples as well, as demonstrated in the meta-analysis of ADHD PGSs across several studies [3]. Examining PGSs in population-based cohorts like PNC will enhance our understanding of the neurogenetic basis of ADHD in a typically developing sample. We specifically examined whether brain volumes in five regions of interests (i.e., caudate, cingulate gyrus, angular gyrus, DLPFC, and inferior temporal lobe) will statistically mediate the association between PGSs and ADHD. PGSs were computed in two ways, one as a “traditional” PGS (i.e., the clumping/pruning + thresholding method [10]) and another that leverages functional annotations of genetic variants from a GWA study of ADHD via *AnnoPred* [11]. First, we hypothesized that both the traditional and *AnnoPred* PGS will associate with ADHD outcomes (i.e., HI and IA symptoms) across age groups. Second, and based on previously reported age-related brain volume differences between ADHD cases and controls across age groups, we hypothesized that the volumes of all five brain regions will mediate the associations between the *AnnoPred* ADHD PGS (given that that it is a more biologically-informed measure of polygenic liability over the traditional ADHD PGS) and ADHD outcomes in children and adolescents, but not in adults. We made no hypothesis regarding potential genetic differences for ADHD IA and HI symptoms given that there have been no strong lines of evidence suggesting differential ADHD PGS associations by ADHD presentation type.

## Method

### Participants

The Philadelphia Neurodevelopmental Cohort (PNC) is a population-based cohort of children, adolescents, and young adults with data collected on psychiatric disorders, medical history, neuroimaging, genetics, and neurocognition. Participants were recruited from the greater Philadelphia, Pennsylvania, area between November 2009 and December 2011 [32]. Psychiatric disorders were assessed using a semi-structured, computerized clinical interview adapted from the Kiddie Schedule for Affective Disorders and Schizophrenia (K-SADS). The interview was administered to caregivers or legal guardians (i.e., collaterals) for participants aged 8 to 10 (i.e., children; analytic *n* = 137), to participants and collaterals for participants aged 11 to 17 (i.e., adolescent; analytic *n* = 297), and to participants themselves if they were between the ages of 18 and 21 (i.e., young adults; analytic *n* = 121). To ensure that there was some degree of consistency with respect to raters, we used data from collaterals for participants between 8 and 17 years of age and from self-report for those older than 18. For additional details on phenotyping and coding, please refer to He and Li [33]. Due to known population stratification effects in ad-mixed samples and the fact that many PGSs are highly underpowered to predict outcomes in non-European ancestry populations [34, 35], we focused the current analyses on PNC individuals who self-reported as European ancestry with genotypic, phenotypic, and neuroimaging data (after quality control), resulting in a total analytic *N* of 555.

### Brain imaging and data processing

Neuroimaging details for the PNC sample are provided in Sattherwaite et al. [32] and are briefly summarized here. Data were acquired from a 3T Siemens TIM Trio scanner at the University of Pennsylvania. The structural images used for this study were obtained via a magnetization-prepared 180° radio-frequency pulses and rapid gradient-echo (MPRAGE) sampling sequence (TR = 1810 ms, TE = 3.5 ms, 160 1 mm slices).

Freesurfer (v. 7.1.1) [36] was used to automatically parcellate and segment the five regions of interest (ROI) used in the study (caudate, cingulate gyrus, angular gyrus, dorsolateral prefrontal cortex, and inferior temporal lobe). These regions were pre-selected based on previous evidence of enrichment for ADHD genetic associations [3, 6]. The command *recon_all.*, with all default parameters taken, was used with processing steps briefly summarized here; the processing stream performs the following sequential steps: skull stripping, volumetric labeling, intensity normalization, white matter segmentation, surface atlas registration, surface extraction, and gyral labeling. The *Destrieux* atlas [37] was used to extract gray matter volume estimates from ROIs by hemisphere. Bilateral ROI volume were used as the mediators, per empirical precedent [38]. To account for the influence of head motion during a scan on the neuroimaging data quality, Euler Numbers for cortical surfaces were extracted by hemisphere from Freesurfer. We averaged across both hemispheres and used the bilateral average Euler Numbers as a covariate in all analyses, per empirical precedent [39].

### ADHD symptoms

IA and HI ADHD symptoms were assessed from six and three dichotomous items (yes/no responses) administered on the adapted K-SADS, respectively. Example items included: “Did you often have trouble paying attention or keeping your mind on your school, work, chores, or other activities that you were doing?”, “Did you often have trouble making plans, doing things that had to be done in a certain kind of order, or that had a lot of different steps?”, and “Did you often blurt out answers to other people’s questions before they finished speaking or interrupt people abruptly?”. Symptom counts of the two ADHD dimensions were computed by summing the number of endorsed items for each dimension.

### Genotyping and polygenic scores

#### *Genotyping in* PNC

All participants were genotyped upon consent using common single nucleotide polymorphism (SNP) arrays including Affymetrix Affy60 and Axiom, and Illumina HumanHap550 (v1, v3), Human610-Quad (v1), and HumanOmniExpress. We applied pre-imputation quality control measures to our genotype data, including removing SNPs with minor allele frequencies (MAF) < 5%, Hardy–Weinberg equilibrium *p* values < 1.0e − 4, and call rates < 95%. We phased and imputed the genotype data using the Haplotype Reference Consortium reference panel version r1.1 2016 available on the Michigan Imputation server [40]. After imputation, we further removed duplicated and strand-ambiguous SNPs, as well as SNPs with MAF < 0.01 or an imputation quality score below 0.8. Samples genotyped using different arrays were processed in separate batches.

#### Discovery GWA study

The ADHD GWA study was a case–control meta-analysis that consisted of 55,374 children and adults (20,183 cases and 35,191 controls) from 12 studies of mixed (but predominantly European) ancestries [6]. The largest cohort among these 12 studies was a population-based case–control cohort in Denmark (iPSYCH; 14,584 cases and 22,492 controls). The other 11 case–control or trio samples were collected in Europe, Canada, USA, and China and aggregated by the Psychiatric Genomic Consortium. ADHD case status was determined based on International Classification of Diseases, tenth revision (ICD-10) in iPSYCH, and semi-structured clinical interviews (e.g., Schedule for Affective Disorders and Schizophrenia for School-Age Children, K-SADS) in the other 11 cohorts.

#### *Traditional PGS (i.e., clumping/pruning* + *thresholding)*

To construct the traditional PGS, we used PLINK [41] to clump ADHD GWA study summary statistics and used the European ancestry samples in the 1000 Genomes Project Phase III cohort [42] as the linkage disequilibrium (LD) reference panel. We then specified an LD window size of 1000 kb and a *r*^2^ threshold of 0.1 for clumping. Finally, we generated an ADHD PGS in the PNC target sample using *PRSice-2* software [43] and specified a *p* value threshold of 1.0, which allowed us to include all available SNP information while also facilitating the generalizability of our findings to other samples.

#### AnnoPred PGS

We also constructed a PGS for ADHD using *AnnoPred* [11], a Bayesian framework that leverages genomic annotation information to improve polygenic risk prediction. This approach improves SNP effect size estimation by integrating publicly available ADHD GWA study data [6] and ADHD heritability enrichment in various functional annotation categories estimated using linkage disequilibrium score regression (LDSC) [44]. We incorporated 53 baseline annotations in LDSC [44], GenoCanyon annotation quantifying the overall genomic functionality [45], and 66 GenoSkylinePlus cell-type specific annotations [46] to improve the ADHD *AnnoPred* score. We used 1000 Genomes Project European samples [42] as the linkage disequilibrium reference and the infinitesimal prior in *AnnoPred* to produce SNP posterior mean effects.

#### Analyses

We fit parallel multiple mediation models that included brain volumes of five ROIs (caudate, cingulate gyrus, angular gyrus, DLPFC, and inferior temporal lobe) as mediators, ADHD PGSs (traditional and *AnnoPred*) as predictors, and ADHD IA and HI symptoms as outcomes. In statistical mediation, the bivariate association between the predictor and the outcome is termed a *total* effect. The association between the predictor and the outcome via mediators is termed the *indirect* effect. Finally, the association between the predictor and the outcome after accounting for the mediators is termed the *direct* effect [47]. Parallel multiple mediation models were conducted to account for expected correlations among the volumes of each of the five brain regions. Given the number of models and tests conducted, *p* values were false discover rate (FDR) adjusted using the Benjamin-Hochberg method [48]^1^. In age-stratified analyses, we divided the sample by age groups (8–11-year-olds were labeled as “children,” 12–17-year-olds were “adolescents,” and 18–21-year-olds were “young adults”) to test age-specific effects of PGSs and indirect effects of PGSs via the volumes of the five brain regions. Model fit was evaluated using the Comparative Fit Index (“good” =  > .95) and the root mean square error of approximation (“good” =  < .06) [49]. Analyses were conducted in R *4.1.2* using the following packages: *semTools*,* lavaan*,* psych*, and *stats*. Age, biological sex, standardized total intracranial volume, Euler Numbers, and the first 10 genetic principal components to account for population stratification were included as covariates in all models. It should be noted that covarying out genetic principal components does not correct for all population stratification effects, however [50].

## Results

### Descriptive statistics

The average age of the PNC sample was 14.36 (*sd* = 3.39), with 272 (49.10%) female participants. When stratified by age groups (i.e., children, adolescents, and young adults), the average age was 9.672 (*sd* = 1.058), 14.790 (*sd* = 1.710), and 18.64 (*sd* = 0.837), respectively. Furthermore, 46.715% (*n* = 64), 46.464% (*n* = 138), and 57.851% (*n* = 70) were females across these age groups, respectively. Participants had an average of 2.489 (*sd* = 3.080), 2.037 (*sd* = 2.883), and 2.380 (*sd* = 2.675) ADHD symptoms, respectively. More details of the sample are shown in Table 1, including standardized (in the full sample) regional brain volume and total intracranial volume.

**Table 1:** Descriptive statistics

	Full	Children	Adolescents	Young adults
*N*	555	137	297	121
Age (*sd*)	14.360 (3.394)	9.672 (1.058)	14.790 (1.710)	18.640 (.837)
Biological sex (# female/percentage)	272 (49.100%)	64 (46.715%)	138 (46.464%)	70 (57.851%)
ADHD total symptoms (*sd*)	2.223 (2.891)	2.489 (3.080)	2.037 (2.883)	2.380 (2.675)
ADHD inattention symptoms (*sd*)	1.634 (2.173)	1.759 (2.235)	1.562 (2.249)	1.669 (1.908)
ADHD hyperactivity/impulsivity symptoms (*sd*)	.589 (.967)	.729 (1.068)	.475 (.893)	.711 (.995)
Brain regions (volume)				
Angular gyrus, standardized (*sd*)	− .010 (.867)	.458 (.872)	− .049 (.803)	− .044 (.754)
Caudate, standardized (*sd*)	− .011 (.975)	.302 (.935)	− .068 (.987)	− .227 (.909)
Cingulate gyrus, standardized (*sd*)	− .013 (.988)	.469 (.874)	− .044 (.984)	− .485 (.872)
DLPFC, standardized (*sd*)	− .015 (.983)	.547 (.934)	− .073 (.934)	− .511 (.906)
Inferior temporal lobe, standardized (*sd*)	− .008 (.996)	.328 (.890)	.022 (.947)	− .460 (1.062)
Total intracranial volume, mm^3^ (*sd*)	1413671 (143764.2)	1417708 (114153.5)	1421687 (146137.3)	1389422 (164925.8)

### Traditional ADHD PGS—full sample

**Fig. 1:**
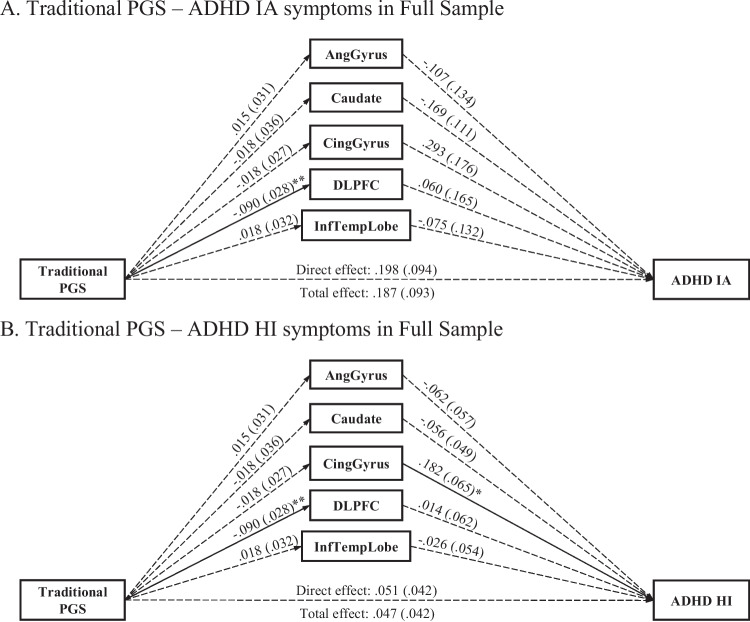
Multiple mediation paths between traditional ADHD PGS and ADHD symptoms via five brain regions in the full sample of youths and young adults in PNC

Multiple mediation models for IA and HI symptoms fit the data well (CFI = .995, RMSEA = .096 for both models). We found no significant total and direct effects of the traditional ADHD PGS on either HI or IA symptoms (see Fig. 1; *FDR*-corrected *p* > .05). Furthermore, no indirect effects emerged with the traditional ADHD PGS on HI or IA symptoms via the volumes of angular gyrus, caudate, cingulate gyrus, DLPFC, and the inferior temporal lobe. However, the traditional ADHD PGS was negatively associated with DLPFC volume (*b* =  − .090, *se* = .028, *FDR*-corrected *p* = .002). In addition, cingulate gyrus volume was positively associated with HI symptoms (*b* = .182, *se* = .065, *FDR*-corrected *p* = .020).

### Traditional ADHD PGS—age-stratified models

**Fig. 2:**
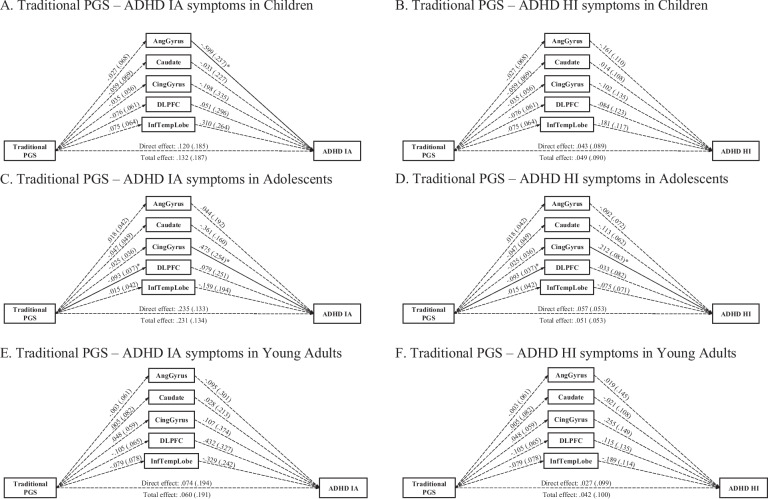
Multiple mediation paths between traditional ADHD PGS and ADHD symptoms via brain regions in children, adolescents, and adults in PNC

The same models were tested separately in each of the three age groups to investigate the possibility of developmental differences of the traditional ADHD PGS on IA and HI symptoms, and the possible mediational effects via the volumes of angular gyrus, caudate, cingulate gyrus, DLPFC, and the inferior temporal. For children (*n* = 137), the models fit well for IA and HI symptoms (CFI = .999, RMSEA = .042 for both models). No total, direct, or indirect effects via the volumes of the five brain regions were detected (Fig. 2). The traditional ADHD PGS was also not associated with any of the five brain volumes. However, the angular gyrus volume was negatively associated with IA symptoms (*b* =  *− *.599, *se* = .237, *FDR*-corrected *p* = .024).

Similarly, no total or direct effects of the traditional ADHD PGS nor indirect effects via the volumes of the five brain regions were detected for adolescents *(n* = 297) (Fig. 2). The models for IA and HI symptoms fit acceptably (CFI = .992, RMSEA = .118 for both models). The traditional ADHD PGS was negatively associated with DLPFC volume (*b* = -.093, *se* = .037, *FDR*-corrected *p* = .024). Additionally, cingulate gyrus volume was positively associated with HI symptoms (*b* = .212, *se* = .083, *FDR*-corrected *p* = .040), and caudate volume was negatively associated with IA symptoms (*b* =  *− *.361, *se* = .160, *FDR*-corrected *p* = .048).

For young adults, the models fit well for both IA and HI symptoms (CFI = .998, RMSEA = .032 for both models). We observed no total or direct effects of the traditional PGS nor indirect effects of the traditional PGS via the volumes of the five brain regions in young adults (see Fig. 2). In addition, no associations were detected between the traditional ADHD PGS and brain volumes, nor between any of the brain volumes and HI or IA symptoms (see Fig. 2).

### AnnoPred ADHD PGS—full sample

**Fig. 3:**
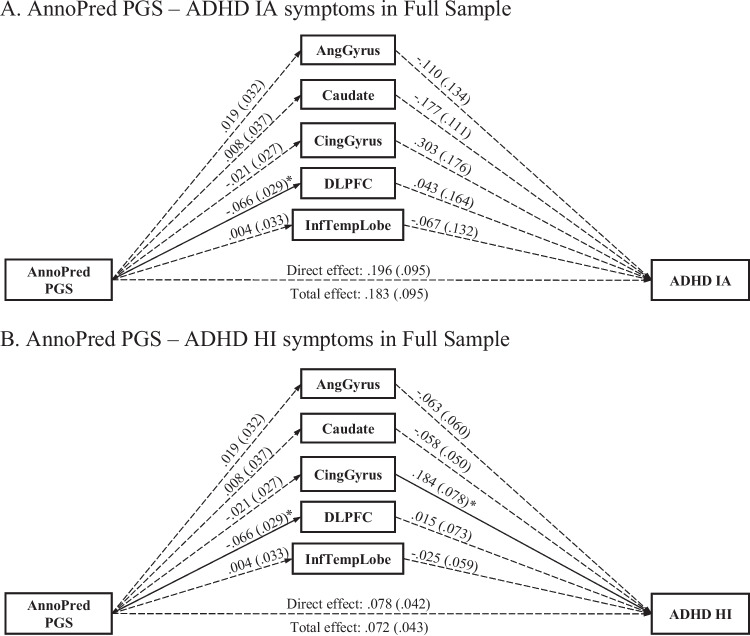
Multiple mediation paths between AnnoPred ADHD PGS and ADHD symptoms via brain regions in the full sample of children, adolescents, and young adults in PNC

The models for IA and HI symptoms fit well (CFI = .995, RMSEA = .098 for both models). There was neither a total nor direct effect of the *AnnoPred* ADHD PGS on HI or IA symptoms (Fig. 3). There was also no total indirect effect of the *AnnoPred* ADHD PGS on IA or HI symptoms via the volumes of the five brain regions. However, the *AnnoPred* ADHD PGS was negatively associated with DLPFC volume (*b* =  *− *.066, *se* = .029, *FDR*-corrected *p* = .022). Additionally, cingulate gyrus volume was positively associated with HI symptoms (*b* = .184, *se* = .078, *FDR*-corrected *p* = .038).

### AnnoPred ADHD PGS—age-stratified models

**Fig. 4:**
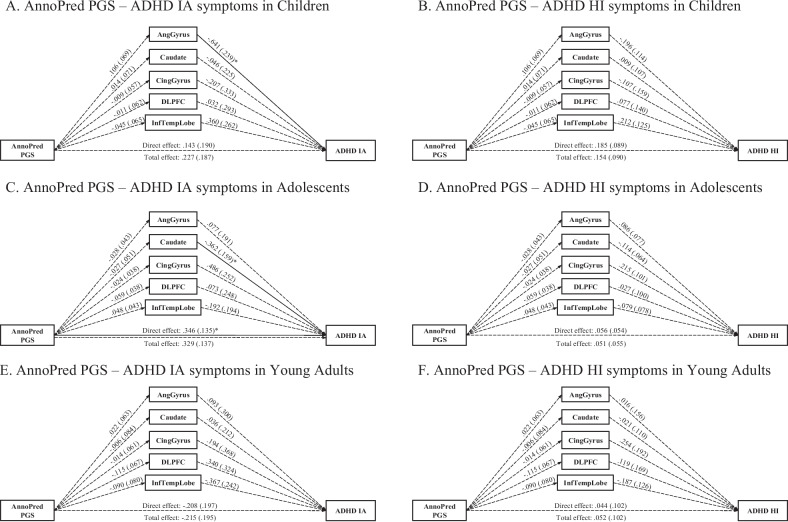
Multiple mediation paths between AnnoPred ADHD PGS and ADHD symptoms via brain regions in children, adolescents, and young adults in PNC

For children, the models fit well for IA and HI symptoms (CFI = .998, RMSEA = .051 for both models). Total and direct effects of the *AnnoPred* ADHD PGS on HI and IA symptoms were not significant, nor were the indirect effects via the volumes of five brain regions (Fig. 4). The *AnnoPred* ADHD PGS was also not associated with any of the volumes of the five brain regions. However, angular gyrus volume was negatively associated with IA symptoms (*b* =  *− *.641, *se* = .239, *FDR*-corrected *p* = .024).

For adolescents, the models fit acceptably for IA and HI symptoms (CFI = .992, RMSEA = .118 for both models). There was a direct effect of the *AnnoPred* ADHD PGS on IA symptoms (*b* = .346, *se* = .135, *FDR*-corrected *p* = .044), but not on HI symptoms. No total or indirect effects of the *AnnoPred* ADHD PGS emerged for IA or HI symptoms via the five brain volumes (Fig. 4). The *AnnoPred* ADHD PGS was also not associated with any of the five brain volumes. However, caudate volume was negatively associated with IA symptoms (*b* =  − .362, *se* = .159, *FDR*-corrected *p* = .048).

Finally, for young adults the models fit well for IA and HI symptoms (CFI = .999, RMSEA = .032 for both models). We detected no significant total or direct effect of the *AnnoPred* ADHD PGS, nor were there significant indirect effects of the *AnnoPred* ADHD PGS via the volumes of five brain regions (Fig. 4). No association was detected between the *AnnoPred* ADHD PGS and brain volumes, nor between brain volumes with IA and HI symptoms (Fig. 4).

## Conclusions

The primary objective of this study was to investigate whether associations between ADHD outcomes and two types of ADHD PGSs, one computed using a traditional method and another that was biologically informed using the *AnnoPred* method, would be mediated by volumes of five brain regions—the caudate, cingulate gyrus, angular gyrus, DLPFC, and inferior temporal lobe—in a population-based dataset of children, adolescents, and young adults. We also examined age-stratified effects of the potential mediation pathways between ADHD PGSs and ADHD symptoms. With respect to our first hypothesis, we found that the traditional ADHD PGS was not associated with either IA or HI symptoms across all developmental age groups. However, the *AnnoPred* ADHD PGS was associated with IA symptoms, but only in adolescents. With respect to our second hypothesis, brain volumes of the caudate, cingulate gyrus, angular gyrus, DLPFC, and the inferior temporal lobe did not statistically mediate the association between the *AnnoPred* ADHD PGS on IA and HI ADHD symptoms. This was consistent in our mediation models in the full sample and in the age-stratified models.

First, we found that the *AnnoPred* ADHD PGS was associated with IA (but not HI) symptoms for adolescents specifically. In contrast, the traditional ADHD PGS was not associated with ADHD outcomes in any of our models. Incorporating biological information into PGS computations may yield more developmentally sensitive predictive signals for ADHD symptoms than traditional ADHD PGSs, which have previously been shown to be less discriminative between various age groups [3, 9]. One reason is that there may be distinct genetic influences for ADHD that depend on the age and developmental stage of the individual [1, 51–54]. This possibility is supported by evidence from another ADHD GWA study, which found distinct genetic architectures underlying childhood ADHD, persistent ADHD, and late-diagnosed ADHD [55]. Our finding also supports the possibility that the genetic liability for ADHD may be developmentally specific, such that the *AnnoPred* ADHD PGS was only associated with IA but not HI symptoms in adolescence, which is consistent with the relative stability of IA symptomology over time in individuals [27].

Our findings did not support our second hypothesis that the association between *AnnoPred* ADHD PGS and ADHD symptoms would be mediated by brain volumes. In fact, the lack of strong associations between the *AnnoPred* ADHD PGS and the volumes of caudate, cingulate gyrus, angular gyrus, DLPFC, and the inferior temporal lobe, and between the volumes of these five brain regions and ADHD symptoms may be due to the non-clinical population of the PNC dataset. The relative lack of variability in ADHD symptomology in the PNC dataset may have limited our power to detect strong effect sizes in our study. In addition, most structural MRI studies of ADHD and ADHD GWA were conducted with case–control samples of youths and/or adults [56]. The brain regions we focused on in our study were informed by these ADHD case–control studies, which may not have been generalizable to large population-based studies [57]. Future studies should examine the possibility that neurogenetic mechanisms of ADHD may differ between clinical and the general population samples [58].

However, we did detect a negative association between both the traditional and *AnnoPred* ADHD PGSs and DLPFC volumes. Although DLPFC volume was not associated with ADHD symptoms perhaps due to the limited variability in ADHD symptoms in the PNC sample, prior studies have found that the DLPFC was the most enriched region with respect to ADHD-associated genetic variants relative to the other brain regions we tested [6, 19]. In our age-stratified analyses, we also observed a negative association between the traditional ADHD PGS and DLPFC volume in adolescents specifically, which might be attributable to an increased heritability of DLPFC volume from childhood to adolescence [59, 60]. Given that our study was cross-sectional, future studies should explore changes in the heritability of brain measures in relation to the development of ADHD.

Additionally, we found a positive association between cingulate gyrus volume and HI symptoms in the full sample and in adolescents specifically. This finding was inconsistent from prior literature which found reduced volume in the cingulate cortex in youths and adults with ADHD relative to controls [15, 56, 61]. The cingulate gyrus is known for its role in controlled cognition, response inhibition, novelty detection, and motivation [61]. We posit two possible explanations for our findings. First, previous studies regressed the volume of each region of interests separately on ADHD status, along with a set of covariates (e.g., biological sex, age, total intracranial volume). Our study examined the cingulate simultaneously with several other brain regions within a multiple mediator framework (in addition to accounting for other covariates), thereby accounting for the high degree of covariation between these brain regions. Additionally, previous studies were conducted in clinical and/or case–control samples. The cingulate cortex along with other previously implicated brain regions for ADHD may associate with natural variations in ADHD symptoms differently in population-based samples that feature lesser severity and greater heterogeneity of ADHD presentations.

Our study was limited by several noteworthy issues. First, our measure of ADHD in young adults was based on retrospective self-reports, whereas ADHD was measured in children and adolescents using collateral (i.e., parent) reports. Reporter heterogeneity [62] may have affected the generalizability/comparability of our findings between children/adolescents and young adults in our study. Relatedly, self-reported measures of ADHD could be related to lower heritability estimates relative to ADHD reported by other informants [63]. Second, medication statuses were unknown, thus precluding our ability to test whether our findings were robust against the effect of medication on both ADHD symptoms and on cortical maturation. Third, ADHD IA and HI symptoms were measured by only six and three items respectively, thus limiting the variability of total ADHD symptoms in the sample. It is possible that a lack of variance in ADHD HI symptoms (in particular) could have driven the developmental specificity of *AnnoPred* PGS and IA symptoms in adolescents, but not for HI symptoms. Fourth, we examined cortical and subcortical volumes in our study, in line with empirical precedent [38]. However, other brain structural measures (e.g., cortical thickness, surface area, fractional anisotropy) as well as functional measures (e.g., regional and network activation) have been studied in relation to ADHD but were not tested in the current study [15, 56, 57, 64]. Future studies may consider incorporating other brain measures to test neurogenetic mechanisms of ADHD. Fifth, although the current study was well-powered and our sample size was comparatively larger than many previous structural MRI studies of ADHD [18, 65], recent studies suggest that it may require several thousands of individuals in the sample to detect reliable and robust associations between brain volumes and complex behaviors such as ADHD symptoms [57, 66]. The smaller effect sizes we reported and the lack of statistical significance in many hypothesized associations, therefore, should be interpreted with some caution. Sixth, PNC is a cross-sectional study. The absence of repeated measures precluded our ability to examine whether (or how) genetic variants may impact both brain structures and ADHD symptomology over time. Finally, our analyses were limited to PNC individuals of self-reported European ancestry. It has been well documented that PGSs lack predictive portability across non-European ancestries [35] given that non-European GWA discovery sample sizes are comparatively small and underpowered relative to European sample sizes (e.g., Duncan et al., [67]). Additionally, others have noted that trans-ancestral PGS predictions may misrepresent the true association between genetic risk and a wide range of phenotypes [35, 68, 69]. Clearly, more diverse samples are needed to better address the growing racial-ethnic disparity in psychiatric genetics research.

To conclude, we found a developmentally specific direct effect of the *AnnoPred* ADHD PGS on IA symptoms, suggesting the utility of leveraging functional annotation information in computations of ADHD PGSs. Still, the “average” ADHD PGS explains only about 4% of the variance in ADHD outcomes across studies, and even less variance in population-based cohorts [3]. As GWA study sample sizes continue to climb, ADHD PGSs should become more powerful predictors over time. Including functional annotation information into PGS computations may not only yield more powerful prediction signals for ADHD, but it may also help to elucidate brain-based biomarkers underlying the genetic risk for ADHD which will be critical for the development of novel therapeutics.

## Data Availability

The data used in this study are publicly available in dbGaP (https://www.ncbi.nlm.nih.gov/gap/). Study scripts and codebooks to query and process the symptom-level data can be accessed on our study preregistration via the Open Science Framework: https://osf.io/whjuq

## Abbreviations

ADHD: Attention-deficit/hyperactivity disorder
CFI: Comparative Fit Index
FRD: False discovery rate
DLPFC: Dorsolateral prefrontal cortex
GWA: Genome-wide association
HI: Hyperactivity-impulsivity
IA: Inattention
LDSC: Linkage disequilibrium score regression
MAF: Minor allele frequency
PGS: Polygenic score
PNC: Philadelphia Neurodevelopmental Cohort
RMSEA: Root Mean Square Error of Approximation
ROI: Region of interest
SNP: Single-nucleotide polymorphism

## Footnotes

1 For clarity, we refer to the *p* values as “FDR-adjusted *p values*”.

## References

[1] Moffitt TE, Houts R, Asherson P, Belsky DW, Corcoran DL, Hammerle M (2015). Is adult ADHD a childhood-onset neurodevelopmental disorder? Evidence from a four-decade longitudinal cohort study. AJP.

[2] Polanczyk GV, Salum GA, Sugaya LS, Caye A, Rohde LA (2015). Annual research review: a meta-analysis of the worldwide prevalence of mental disorders in children and adolescents. J Child Psychol Psychiatry.

[3] Li JJ, He Q. Polygenic scores for ADHD: a meta-analysis. Res Child Adolesc Psychopathol. 2021. Available from: 10.1007/s10802-021-00774-4. Cited 2021 Jan 25. 10.1007/s10802-021-00774-4 33492530

[4] Demontis D, Walters RK, Rajagopal VM, Waldman ID, Grove J, Als TD (2021). Risk variants and polygenic architecture of disruptive behavior disorders in the context of attention-deficit/hyperactivity disorder. Nat Commun.

[5] Faraone SV, Larsson H (2018). Genetics of attention deficit hyperactivity disorder. Mol Psychiatry.

[6] Demontis D, Walters RK, Martin J, Mattheisen M, Als TD, Agerbo E (2019). Discovery of the first genome-wide significant risk loci for attention deficit/hyperactivity disorder. Nat Genet.

[7] Demontis D, Walters GB, Athanasiadis G, Walters R, Therrien K, Farajzadeh L, et al. Genome-wide analyses of ADHD identify 27 risk loci, refine the genetic architecture and implicate several cognitive domains. medRxiv. 2022: 2022.02.14.22270780. Available from: https://www.medrxiv.org/content/10.1101/2022.02.14.22270780v1.Cited 2022 Jun 22. 10.1038/s41588-022-01285-8 PMC10914347 36702997

[8] Plomin R, Haworth CMA, Davis OSP (2009). Common disorders are quantitative traits. Nat Rev Genet.

[9] Ronald A, de Bode N, Polderman TJC (2021). Systematic review: how the attention-deficit/hyperactivity disorder polygenic risk score adds to our understanding of ADHD and associated traits. J Am Acad Child Adolesc Psychiatry.

[10] Purcell SM, Wray NR, Stone JL, Visscher PM, O’Donovan MC, Sullivan PF (2009). Common polygenic variation contributes to risk of schizophrenia and bipolar disorder. Nature.

[11] Hu Y, Lu Q, Powles R, Yao X, Yang C, Fang F (2017). Leveraging functional annotations in genetic risk prediction for human complex diseases. PLoS Comput Biol.

[12] Tost H, Bilek E, Meyer-Lindenberg A (2012). Brain connectivity in psychiatric imaging genetics. Neuroimage.

[13] Meyer-Lindenberg A, Weinberger DR (2006). Intermediate phenotypes and genetic mechanisms of psychiatric disorders. Nat Rev Neurosci.

[14] Castellanos FX, Lee PP, Sharp W, Jeffries NO, Greenstein DK, Clasen LS (2002). Developmental trajectories of brain volume abnormalities in children and adolescents with attention-deficit/hyperactivity disorder. JAMA.

[15] Kobel M, Bechtel N, Specht K, Klarhöfer M, Weber P, Scheffler K (2010). Structural and functional imaging approaches in attention deficit/hyperactivity disorder: does the temporal lobe play a key role?. Psychiatry Res.

[16] Krain AL, Castellanos FX (2006). Brain development and ADHD. Clin Psychol Rev.

[17] Sowell ER, Thompson PM, Welcome SE, Henkenius AL, Toga AW, Peterson BS (2003). Cortical abnormalities in children and adolescents with attention-deficit hyperactivity disorder. Lancet.

[18] Frodl T, Skokauskas N (2012). Meta-analysis of structural MRI studies in children and adults with attention deficit hyperactivity disorder indicates treatment effects. Acta Psychiatr Scand.

[19] Kasparek T, Theiner P, Filova A (2015). Neurobiology of ADHD from childhood to adulthood: findings of imaging methods. J Atten Disord.

[20] Seidman LJ, Valera EM, Makris N, Monuteaux MC, Boriel DL, Kelkar K (2006). Dorsolateral prefrontal and anterior cingulate cortex volumetric abnormalities in adults with attention-deficit/hyperactivity disorder identified by magnetic resonance imaging. Biol Psychiat.

[21] Vilgis V, Sun L, Chen J, Silk TJ, Vance A (2016). Global and local grey matter reductions in boys with ADHD combined type and ADHD inattentive type. Psychiatry Res.

[22] Grahn JA, Parkinson JA, Owen AM (2008). The cognitive functions of the caudate nucleus. Prog Neurobiol.

[23] Hertrich I, Dietrich S, Blum C, Ackermann H (2021). The role of the dorsolateral prefrontal cortex for speech and language processing. Front Human Neurosci.

[24] Buckner RL (2013). The brain’s default network: origins and implications for the study of psychosis. Dialogues Clin Neurosci.

[25] Liddle EB, Hollis C, Batty MJ, Groom MJ, Totman JJ, Liotti M (2011). Task-related default mode network modulation and inhibitory control in ADHD: effects of motivation and methylphenidate. J Child Psychol Psychiatry.

[26] Alemany S, Jansen PR, Muetzel RL, Marques N, El Marroun H, Jaddoe VWV (2019). Common polygenic variations for psychiatric disorders and cognition in relation to brain morphology in the general pediatric population. J Am Acad Child Adolesc Psychiatry.

[27] Franke B, Michelini G, Asherson P, Banaschewski T, Bilbow A, Buitelaar JK (2018). Live fast, die young? A review on the developmental trajectories of ADHD across the lifespan. Eur Neuropsychopharmacol.

[28] Holbrook JR, Cuffe SP, Cai B, Visser SN, Forthofer MS, Bottai M (2016). Persistence of parent-reported ADHD symptoms from childhood through adolescence in a community sample. J Atten Disord.

[29] Pingault JB, Viding E, Galéra C, Greven CU, Zheng Y, Plomin R (2015). Genetic and environmental influences on the developmental course of attention-deficit/hyperactivity disorder symptoms from childhood to adolescence. JAMA Psychiat.

[30] Hoogman M, Bralten J, Hibar DP, Mennes M, Zwiers MP, Schweren LSJ (2017). Subcortical brain volume differences in participants with attention deficit hyperactivity disorder in children and adults: a cross-sectional mega-analysis. Lancet Psychiatry.

[31] Greven CU, Bralten J, Mennes M, O’Dwyer L, van Hulzen KJE, Rommelse N (2015). Developmentally stable whole-brain volume reductions and developmentally sensitive caudate and putamen volume alterations in those with attention-deficit/hyperactivity disorder and their unaffected siblings. JAMA Psychiat.

[32] Satterthwaite TD, Connolly JJ, Ruparel K, Calkins ME, Jackson C, Elliott MA (2016). The philadelphia neurodevelopmental cohort: a publicly available resource for the study of normal and abnormal brain development in youth. Neuroimage.

[33] He Q, Li JJ (2021). Factorial invariance in hierarchical factor models of mental disorders in African American and European American youths. J Child Psychol Psychiatry.

[34] Duncan L, Shen H, Gelaye B, Meijsen J, Ressler K, Feldman M (2019). Analysis of polygenic risk score usage and performance in diverse human populations. Nat Commun..

[35] Martin AR, Kanai M, Kamatani Y, Okada Y, Neale BM, Daly MJ (2019). Clinical use of current polygenic risk scores may exacerbate health disparities. Nat Genet.

[36] Fischl B (2012). FreeSurfer. Neuroimage.

[37] Destrieux C, Fischl B, Dale A, Halgren E (2010). Automatic parcellation of human cortical gyri and sulci using standard anatomical nomenclature. Neuroimage.

[38] Das D, Cherbuin N, Anstey KJ, Abhayaratna W, Easteal S (2017). Regional brain volumes and ADHD symptoms in middle-aged adults: the PATH through life study. J Atten Disord.

[39] Rosen AFG, Roalf DR, Ruparel K, Blake J, Seelaus K, Villa LP (2018). Quantitative assessment of structural image quality. Neuroimage.

[40] Das S, Forer L, Schönherr S, Sidore C, Locke AE, Kwong A (2016). Next-generation genotype imputation service and methods. Nat Genet.

[41] Purcell S, Neale B, Todd-Brown K, Thomas L, Ferreira MAR, Bender D (2007). PLINK: a tool set for whole-genome association and population-based linkage analyses. Am J Hum Genet.

[42] Auton A, Abecasis GR, Altshuler DM, Durbin RM, Abecasis GR, Bentley DR (2015). A global reference for human genetic variation. Nature.

[43] Choi SW, O’Reilly PF (2019). PRSice-2: polygenic risk score software for biobank-scale data. Gigascience.

[44] Finucane HK, Bulik-Sullivan B, Gusev A, Trynka G, Reshef Y, Loh PR (2015). Partitioning heritability by functional annotation using genome-wide association summary statistics. Nat Genet.

[45] Lu Q, Hu Y, Sun J, Cheng Y, Cheung KH, Zhao H (2015). A statistical framework to predict functional non-coding regions in the human genome through integrated analysis of annotation data. Sci Rep.

[46] Lu Q, Powles RL, Abdallah S, Ou D, Wang Q, Hu Y (2017). Systematic tissue-specific functional annotation of the human genome highlights immune-related DNA elements for late-onset Alzheimer’s disease. PLoS Genet.

[47] Baron RM, Kenny DA. The moderator-mediator variable distinction in social psychological research: conceptual, strategic, and statistical considerations. J Pers Soc Psychol. 1986;51(6):1173–82. 10.1037//0022-3514.51.6.1173 3806354

[48] Benjamini Y, Hochberg Y (1995). Controlling the false discovery rate: a practical and powerful approach to multiple testing. J Roy Stat Soc: Ser B (Methodol).

[49] Hu LT, Bentler PM (1999). Cutoff criteria for fit indexes in covariance structure analysis: conventional criteria versus new alternatives. Struct Equ Modeling: A Multidiscipl J.

[50] Price AL, Zaitlen NA, Reich D, Patterson N (2010). New approaches to population stratification in genome-wide association studies. Nat Rev Genet.

[51] Eilertsen EM, Gjerde LC, Kendler KS, Røysamb E, Aggen SH, Gustavson K (2019). Development of ADHD symptoms in preschool children: genetic and environmental contributions. Dev Psychopathol.

[52] Kendler KS, Gardner CO, Lichtenstein P (2008). A developmental twin study of symptoms of anxiety and depression: evidence for genetic innovation and attenuation. Psychol Med.

[53] Larsson JO, Larsson H, Lichtenstein P (2004). Genetic and environmental contributions to stability and change of ADHD symptoms between 8 and 13 years of age: a longitudinal twin study. J Am Acad Child Adolesc Psychiatry.

[54] Lewis GJ, Plomin R (2015). Heritable influences on behavioural problems from early childhood to mid-adolescence: evidence for genetic stability and innovation. Psychol Med.

[55] Rajagopal VM, Duan J, Vilar-Ribó L, Grove J, Zayats T, Ramos-Quiroga JA (2022). Differences in the genetic architecture of common and rare variants in childhood, persistent and late-diagnosed attention-deficit hyperactivity disorder. Nat Genet.

[56] Valera EM, Faraone SV, Murray KE, Seidman LJ (2007). Meta-analysis of structural imaging findings in attention-deficit/hyperactivity disorder. Biol Psychiat.

[57] Bernanke J, Luna A, Chang L, Bruno E, Dworkin J, Posner J (2022). Structural brain measures among children with and without ADHD in the Adolescent Brain and Cognitive Development Study cohort: a cross-sectional US population-based study. The Lancet Psychiatry.

[58] Westreich D (2012). Berkson’s bias, selection bias, and missing data. Epidemiology.

[59] Lenroot RK, Schmitt JE, Ordaz SJ, Wallace GL, Neale MC, Lerch JP (2009). Differences in genetic and environmental influences on the human cerebral cortex associated with development during childhood and adolescence. Hum Brain Mapp.

[60] Rommelse N, Buitelaar JK, Hartman CA (2017). Structural brain imaging correlates of ASD and ADHD across the lifespan: a hypothesis-generating review on developmental ASD–ADHD subtypes. J Neural Transm.

[61] Bush G (2011). Cingulate, frontal, and parietal cortical dysfunction in attention-deficit/hyperactivity disorder. Biol Psychiat.

[62] Kooij JJS, Boonstra AM, Swinkels SHN, Bekker EM, de Noord I, Buitelaar JK (2008). Reliability, validity, and utility of instruments for self-report and informant report concerning symptoms of ADHD in adult patients. J Atten Disord.

[63] Brikell I, Kuja-Halkola R, Larsson H (2015). Heritability of attention-deficit hyperactivity disorder in adults. Am J Med Genet B Neuropsychiatr Genet.

[64] Boedhoe PSW, van Rooij D, Hoogman M, Twisk JWR, Schmaal L, Abe Y (2020). Subcortical brain volume, regional cortical thickness, and cortical surface area across disorders: findings from the ENIGMA ADHD, ASD, and OCD working groups. Am J Psychiatry.

[65] Pereira-Sanchez V, Castellanos FX (2021). Neuroimaging in attention-deficit/hyperactivity disorder. Curr Opin Psychiatry.

[66] Marek S, Tervo-Clemmens B, Calabro FJ, Montez DF, Kay BP, Hatoum AS (2022). Reproducible brain-wide association studies require thousands of individuals. Nature.

[67] Duncan L, Shen H, Gelaye B, Meijsen J, Ressler K, Feldman M (2019). Analysis of polygenic risk score usage and performance in diverse human populations. Nat Commun.

[68] Peterson RE, Kuchenbaecker K, Walters RK, Chen CY, Popejoy AB, Periyasamy S (2019). Genome-wide association studies in ancestrally diverse populations: opportunities, methods, pitfalls, and recommendations. Cell.

[69] Khan A, Turchin MC, Patki A, Srinivasasainagendra V, Shang N, Nadukuru R (2022). Genome-wide polygenic score to predict chronic kidney disease across ancestries. Nat Med.

